# Risk Assessment for the Spread of Flavescence Dorée-Related Phytoplasmas from Alder to Grapevine by Alternative Insect Vectors in Germany

**DOI:** 10.3390/microorganisms11112766

**Published:** 2023-11-14

**Authors:** Barbara Jarausch, Anna Markheiser, Wolfgang Jarausch, Sandra Biancu, Sanela Kugler, Miriam Runne, Michael Maixner

**Affiliations:** 1 Julius Kühn Institute, Federal Research Centre for Cultivated Plants, Institute for Plant Protection in Fruit Crops and Viticulture, Geilweilerhof, 76833 Siebeldingen, Germany; anna.markheiser@julius-kuehn.de (A.M.);; 2 RLP AgroScience, Breitenweg 71, 67435 Neustadt an der Weinstrasse, Germany

**Keywords:** *Allygus* spp., *Orientus ishidae*, 16Sr-V phytoplasmas, *Alnus glutinosa*, *Vitis vinifera*, survival, transmission capacity, quantification, phytoplasma titer, minimum inoculation titer

## Abstract

“Flavescence dorée” (FD)-related phytoplasmas are widespread in alder in Germany and their transmission to grapevine represents a high risk for FD outbreaks when the primary vector, *Scaphoideus titanus*, becomes present in the future. Therefore, the potential role of the Deltocephalinae leafhopper species in transmitting FD-related phytoplasmas from alder to grapevine was studied in extensive transmission trials conducted between 2017 and 2020. The transmission capacity of autochthonous *Allygus* spp. and the invasive *Orientus ishidae* captured on infected alder trees was tested under controlled conditions using various test designs, including grouped insects and single-insect studies. The latter experiments were analyzed in terms of survival probability, transmission success and phytoplasma load in the insects, measured by quantitative PCR. A minimum inoculation titer (MIT) required for successful transmission to alder was defined for both *Allygus* spp. and *O. ishidae*. While *Allygus* spp. exhibited slightly better survival on *Vitis vinifera* compared to *O. ishidae,* the latter displayed higher phytoplasma loads and greater transmission success. Although all species were capable of infecting alder seedlings, *O. ishidae* was able to transmit 16SrV-phytoplasmas directly to single grapevines. Infective adults of *O. ishidae* were captured from the beginning of July until the end of August, while *Allygus* spp. were only considered infective towards the end of the season. Thus, *O. ishidae* likely poses a higher risk for FD transmission from alder to grapevine, albeit at a very low level, as only five out of 90 transmission trials to *V. vinifera* were successful.

## 1. Introduction

“Flavescence dorée” (FD) is a threatening disease of grapevine (*Vitis vinifera* L.) associated with phytoplasmas belonging to the 16SrV ribosomal group [1,2], comprising the subgroups 16SrV-C and 16SrV-D [3]. Based on the *map* genetic locus, the subgroups can be further divided into clusters FD1, FD2 and FD3, with FD1 and FD3 including strains from subgroup 16SrV-C and cluster FD2 including strains from both subgroups [4,5]. To date, FD is transmitted from vine to vine by the Neartic leafhopper *Scaphoideus titanus* Ball, introduced into Europe in the first half of the 20th century [6]. *Scaphoideus titanus* is monophagous on *Vitis vinifera* and is the main source for the epidemic spread of the disease within vineyards.

However, it has been shown that other Auchenorryncha species may harbour FD phytoplasmas, e.g., the invasive mosaic leafhopper *Orientus ishidae* (Matsumara) [7] or the Palaearctic *Dictyophara europaea* (Linnaeus), which have been confirmed to transmit FD phytoplasmas from *Clematis vitalba* L. to grapevine [8]. Some years later, the capability of *O. ishidae* to acquire it from an infected source and transmit it to grapevine under laboratory conditions was proven [9].

The “flavescence dorée” phytoplasma epidemiology is not limited to the ‘grapevine–*S.titanus’* pathosystem within the vineyard but may represent a complex system at a broader landscape scale involving additional vectors and reservoir plants [9,10,11,12]. In Europe, in addition to this phytoplasma, other members of the 16SrV group infect grapevine, alder (*Alnus glutinosa* (L.) Gaertn.) and other plant species [5,13]. The 16SrV group phytoplasmas not transmitted by *S. titanus* and infecting alder were referred to as alder yellows phytoplasma (AldY), while those detected in grapevine were called Palatinate grapevine yellows phytoplasma (PGY) [14]. PGY and AldY were found to be genetically closely related to “flavescence dorée” phytoplasmas [15,16]. Further comparative and phylogenetic analyses revealed that these three phytoplasmas were members of the same phylogenetic subclade [3,4]. AldY and PGY were shown to be transmitted from alder to alder and occasionally from alder to grapevine by the Macropsinae leafhopper *Oncopsis alni* Schrank [13,17] without causing epidemic outbreaks. Malembic-Maher et al. [11] demonstrated that none of the PGY-isolates transmitted to *Vicia faba* L. by *Oncopsis alni* were acquired or transmitted by *S. titanus.* In contrast, the Deltocephalinae species *Allygus modestus*/*mixtus* and *O. ishidae* collected from alder were capable of transmitting phytoplasma strains to *Vicia faba* L., which could then be further transmitted by *S.titanus*. The authors elucidated that the “flavescence dorée” phytoplasmas are of European origin and are widespread in natural habitats in hosts like alders. In Germany, alder trees are frequently infected with various 16SrV phytoplasmas, and preliminary studies showed that FD-like phytoplasmas are widespread [18]. Although *S. titanus* has not yet been detected in Germany, a single grapevine infected by “flavescence dorée” phytoplasmas was found in the vicinity of an alder stand and Deltocephalinae species collected from these alders harboured the same phytoplasma strain as the grapevine [19]. During a previous survey of the leafhopper fauna on alders, it was discovered that the autochthonous *Allygus mixtus* (Fabricius) and *Allygus modestus* Scott, as well as the invasive *O. ishidae*, were the most abundant Deltocephalinae species on alder, and they were frequently infected with 16SrV phytoplasmas [20].

The objective of this study was to evaluate the role of *Allygus* spp. and *O. ishidae* in the transmission of “flavescence dorée” phytoplasmas from alder to grapevine and, consequently, to assess the role of these species in the possible spread of this pathogen into vineyards. This issue is of great concern in the context of the possible appearance of *S. titanus* in Germany. In this regard, the transmission capacity of *Allygus* spp. and *O. ishidae* collected from alder in Germany was investigated in more detail. Different types of transmission trials and survival experiments were carried out with these species. Furthermore, their infection status was analyzed, a species-specific minimum titer for successful transmission was defined, and the variation of the phytoplasma load during the season was also assessed. 

## 2. Materials and Methods

### 2.1. Insect and Plant Material

Adult Cicadellidae leafhoppers were collected in different vine-growing areas in southwestern Germany by sweep-netting from infected alder trees adjacent to vineyards, from the beginning of June until September in the years 2017–2020. Specimens of the Deltocephalinae *Allygus modestus*, *Allygus mixtus* and *Orientus ishidae* were collected using a mouth aspirator and transported to the laboratory in cages, where they were either stored at −20 °C or used alive for transmission trials. Dead insects were identified at the species level and sexed based on morphological characteristics according to Kunz et al. [21] and Biedermann and Niedringhaus [22]. As *Allygus* spp. are difficult to distinguish by morphological means, a subset of samples was analyzed by COI barcoding according to the EPPO standard protocol [23], confirming morphological identification results that both *Allygus modestus* and *Allygus mixtus* were present in the samples. 

Alder seedlings, hot water-treated grapevine cuttings of cv. Chardonnay and micropropagated phytoplasma-free plantlets of cv. Scheurebe were grown in 2 L pots at the Julius Kühn-Institute, Siebeldingen, Germany, under insect-proof conditions. Glasshouse conditions were 25  ±  5 °C temperature, 70  ±  10% relative humidity, a photoperiod of 16:8 h light/dark and a minimum of 2500 lx light intensity. 

### 2.2. Transmission Trials

Insects collected in the field from infected *A. glutinosa* were caged on recipient plants either in a dual-choice approach or in single plant assays with or without transfer. The experimental set-up is illustrated in Figure S1a,b (Supplementary Materials).

For dual choice experiments, leafhoppers of the same species (variable group size: 5–20, depending on collections) were caged with seedlings of *A. glutinosa* (8–10 fully developed leaves) and hot-water treated *V. vinifera* cv. Chardonnay test plants (8–10 fully developed leaves) in mesh cages (32.5 cm × 32.5 cm × 32.5 cm, Watkins & Doncaster, Leominster, UK) (Figure S1a) until death. 

In single-plant tests, the insects were caged on potted plants in cylindrical acrylic insect-proof cages covered with a nylon mesh (height 35 cm, diameter 13 cm). For assays with transfer, batches of 5–15 insects were put on alder seedlings (6–10 fully developed leaves) and after an inoculation access period (IAP) of 3–7 days, living specimens were transferred to micropropagated *V. vinifera* plantlets cv. Scheurebe (6–10 fully developed leaves) as second test plants until their death (Figure S1b). Single plant-single insect trials were carried out on seedlings of *A. glutinosa* (6–8 fully developed leaves) and micropropagated *V. vinifera* cv. Scheurebe (6–8 fully developed leaves). 

All tests were carried out under the same conditions in a walk-in climatic chamber Fitotron type SGR233 (Weiss Technik Ltd., Loughborough, UK) at a photoperiod of 16:8 h (1 h each of dusk at dawn), 23:19 ± 2 °C and 75 ± 5% relative humidity. Cylindrical cages were checked daily and dead individuals were removed and stored at −20 °C until final morphological identification and molecular analyses. At the end of the trial, inoculated test plants were removed from the cage, treated once with an insecticide (Spruzit Schädlingsfrei, W. Neudorff GmbH KG, Emmerthal, Germany) and transferred into the glasshouse. Plants were tested for phytoplasma presence three times to confirm successful or failed transmission: (i) approximately 8 weeks after the end of the trial, (ii) after hibernation and dormancy in the cold house (about 6 months after IAP) and (iii) about 12 months after IAP. 

### 2.3. Survival Experiments

In combination with single insect-single plant transmission trials in 2019 and 2020, the survival of *Allygus* spp. and *O. ishidae* specimens on *A. glutinosa* and *V. vinifera* cv. Scheurebe was recorded daily. Cages containing a water source but without any plant served as negative controls. Dead insects were removed from the cages, morphologically identified, sexed and stored at −20 °C until DNA extraction and examination of phytoplasma presence.

### 2.4. DNA Extraction

Total nucleic acid (TNA) of the test plants was extracted from 120 to 150 mg leaf midribs and petioles with the CTAB method by Doyle and Doyle [24], modified according to Boudon-Padieu et al. [25]. The final pellet was resuspended in 150 µL TE buffer. TNA was extracted from healthy plantlets that were raised in the glasshouse with extraction procedures as negative control. 

TNA of insects was extracted according to the CTAB protocol by Maixner et al. [14]. Individuals were homogenized in 250 µL CTAB buffer using a cell mill (TissueLyser II, Qiagen GmbH, Hilden, Germany) with two carbide beads per tube. After precipitation and washing procedures, the final pellet was resuspended in 150 µL TE buffer. 

### 2.5. Phytoplasma Detection

Generic phytoplasma detection was carried out by PCR with the universal primers U5/P7 [26,27], using a 20 µL reaction volume with a final concentration of 0.025 U/µL DreamTaq Green DNA-Polymerase (Thermo Fisher Scientific GmbH, Waltham, MA, USA), 0.2 mM dNTPs, 0.5 µM (each primer), 1X DreamTaq Green Buffer (Thermo Fisher Scientific GmbH, Waltham, MA, USA) and 2 µL template DNA. Cycle conditions were 2 min at 94 °C followed by 5 cycles of (30 s at 94 °C, 30 s at 59 °C and 90 s at 72 °C), 5 cycles of (30 s at 94 °C, 30 s at 58 °C and 90 s at 72 °C), 20 cycles of (30 s at 94 °C, 30 s at 57 °C and 90 s at 72 °C) and a final elongation step of 30 s at 72 °C.

To identify phytoplasmas of the 16SrV ribosomal group, PCR was carried out with the group-specific primers fAY/rEY as described by Maixner and Reinert [17]. 

### 2.6. Amplification and Sequencing of Genetic Markers

PCR-positive samples of insects or inoculated plants were further characterized by amplification of the secY-map locus (*map* gene) by nested PCR and sequencing as described by Arnaud et al. [4] and by the *vmpA* gene marker (*vmp* gene) according to the protocol of Malembic-Maher et al. [11]. Primers and PCR conditions are described in Table S3 in Malembic-Maher et al. [11] The products of both amplifications were purified with the QIAquick PCR Purification Kit (Qiagen GmbH, Hilden, Germany). Sequencing reactions were performed by SeqLab (Microsynth GmbH, Balgach, Switzerland) via Sanger sequencing in both directions on the *map* gene using the primers fFD9F6/rMapR2, and upstream on the *vmpA* gene with primers VMPA-F3/VMPA-R5 [11]. Assembling of raw sequences and multiple alignments were performed with CLC Main Workbench 22.0.2. (Qiagen GmbH, Hilden, Germany) and phylogenetic reconstructions using maximum parsimony were performed by MEGA7 [28] with randomized bootstrapping evaluation.

### 2.7. Phytoplasma Quantification

The phytoplasma concentration in infected insects was determined by quantitative real-time PCR using a newly developed SYBR Green™ assay. The housekeeping gene *map* was used for primer selection. Forward primer MAP U1 (5′-ATCGTTATAATGAAAGAGGC-3′) and reverse primer MAP U2 (5′-TGTTTAATACCTATATCTAAAG-3′) were selected in two conserved regions among the 121 *map* genotypes published by Malembic-Maher et al. [11]. A 272 bp gene fragment of 16SrV phytoplasmas was amplified with these primers (0.22 µM each) using 1× TEMPase Mastermix (VWR International GmbH, Darmstadt, Germany) with 3 mM Mg^2+^ and SYBR Green™ I (Lumiprobe GmbH, Hannover, Germany) diluted to 1:66,000 in 20 µL reactions. Cycle conditions were 15 min at 95 °C for hot start followed by 40 cycles of 15 s at 95 °C, 15 s at 54 °C and 30 s at 72 °C. After the final elongation step of 4 min at 72 °C, a melting curve analysis ranging from 50 °C to 90 °C was performed. Each sample was diluted 1:5 and 1 µL was amplified in duplicate in two independent runs in a Chromo4 Real-Time PCR detector (Bio-Rad Laboratories GmbH, Feldkirchen, Germany). Absolute quantification was performed by the standard curve method using a plasmid containing the *map* sequence of map-genotype M38 in serial 10-fold dilutions ranging from 1 × 10^8^ target copies per µL to 1 copy per µL. The cycle threshold (Ct) values of the standard dilutions were plotted and verified to give a linear relationship, which served as the standard curve. The quality of the standard curve was adjusted manually for each run according to the best linear regression coefficient. Then, the Ct-values of each sample were compared to this standard curve and the copy number in each sample was calculated. The phytoplasma concentrations were normalized to the ng DNA per µL as measured by NanoDrop™ 2000c (Thermo Fisher Scientific GmbH, Waltham, MA, USA).

### 2.8. Statistical Analysis

The effect of the host plant species and/or phytoplasma load of the insect on the survival of *Allygus* spp. and *O. ishidae* was assessed through survival curves, estimated by the Kaplan–Meier method for censored data [29]. A marginal Cox proportional-hazards model was applied where robust standard errors were obtained [30]. The Cox model was validated by checking the proportional hazards assumptions with a Schoenfeld residual analysis [29]. The dependent variable was the survival time of each insect; the categorical explanatory variables were the sex, the phytoplasma infection status, the insect *taxon* (*Allygus* spp. or *O. ishidae*), the transmission success (unsuccessful/successful) and the test plant species (*A. glutinosa*/*V. vinifera*). Pairwise comparisons were performed using Log-rank test with Bonferroni correction. Statistical differences between plants or insect taxa were indicated when *p* < 0.05.

To test the effect of the phytoplasma load on the transmission success to the test plants, a Welch’s t-test was performed for each insect *taxon* as the data violate the assumption of homogeneity of variances. The response variable was the transmission success, and the categorical explanatory variable was the phytoplasma load. 

Seasonal variations of the phytoplasma load within the species *Allygus* spp. and *O. ishidae* were calculated by Welch’s ANOVA, followed by the Games–Howell Post Hoc Test. The response variable was the period within the years, and the categorical explanatory variable was the phytoplasma load. 

All analyses were performed in R version 4.2.2 [31]. Cox models were developed and validated using the ‘survival’ package [32]. Kaplan–Meier curves were plotted using the ‘survminer’ package [33]. Pairwise comparisons were run using the ‘emmeans’ package [34]. Boxplots and barplots were generated with packages ‘ggplot2′ [35] and ‘ggstatsplot’ [36], respectively. 

## 3. Results

### 3.1. Dual-Choice Test

The results of the dual choice trials are summarized in Table 1. In 2017, *O. ishidae* was able to transmit 16SrV-group phytoplasmas from field-grown alder to a high percentage of alder seedlings but was also able to inoculate one grapevine plantlet in the same cage where an alder seedling had been inoculated. In all infected individuals of *O. ishidae* and in all infected test plants (alder and grapevine), the FD-map genotype M38 was identified by sequence analysis. In contrast, in the following year 2018, *Allygus* spp. as well as *O. ishidae* inoculated alder seedlings, but no transmission occurred to *Vitis vinifera* during dual choice experiments. 

### 3.2. Transmission Test with Plant-Transferred Insects

In a second experimental design, insects were initially placed on *A. glutinosa* and then transferred to *V. vinifera*. As depicted in Table 2, a high transmission rate to alder seedlings was observed with both *O. ishidae* and *Allygus* spp. in all years. However, there was no successful inoculation of grapevine plantlets by the same batches of insects that previously inoculated alder. Despite annual fluctuations, infection rates in alders inoculated by *O. ishidae* were consistently higher than those inoculated by *Allygus* spp. This is consistent with the higher infection rate in *O. ishidae* compared to *Allygus* spp., but also with the greater number of *O. ishidae* per test plant. As expected, the infection rates of insects collected from the first test plant (alder) and subsequently from the second test plant (grapevine) were closely matched. Infection rates in *Allygus* spp. showed more variation, with a notable difference in 2019.

### 3.3. Single Insect—Single Plant Transmission Tests

The third experimental setup was designed as single insect-single plant trials and aimed to explore transmission parameters in a one-by-one insect-plant assignment. The results of the transmission trials are summarized in Table 3. The transmission rates to alder in 2019 and 2020 were relatively consistent for *Allygus* spp. (12% vs. 14%), but varied considerably between the years for *O. ishidae* (6% vs. 46%). These variations between years and species are also evident in the transmission efficiency, which represents the proportion of infected leafhoppers that successfully transmitted (*Allygus* spp.: 18% and 19%; *O. ishidae*: 7% and 46%). Nevertheless, individual *O. ishidae* were capable of transmitting the phytoplasma to grapevine with an efficiency of 13%, while there was no successful inoculation by *Allygus* spp. Figure S2 depicts an infected micropropagated plantlet cv. ‘Scheurebe’ three months after inoculation with *O. ishidae*.

### 3.4. Survival Analysis

To assess the suitability of a plant species as feeding host and the influence on the transmission success, we combined the transmission trials with a survival analysis during single insect-single plant assays. In a first step, we verified that there was no significant difference between the two years of the investigation, 2019 and 2020 (*p* > 0.05). Consequently, we pooled the data for further analyses. 

**Figure 1:**
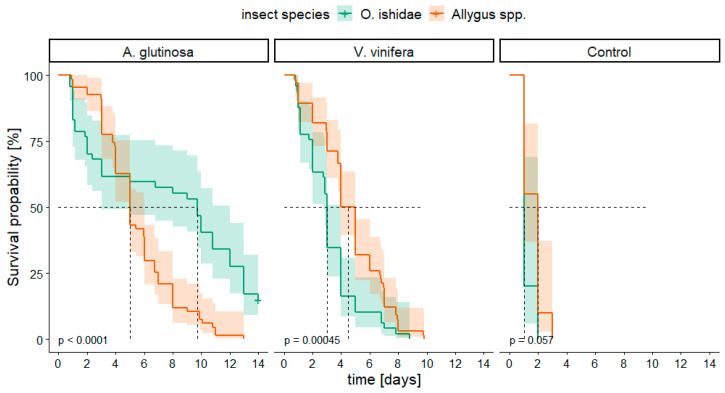
Kaplan–Meier survival curves for *Orientus ishidae* (green; n*_Alnus_* = 47, n*_Vitis_* = 49, n_Control_ = 10) and *Allygus* spp. (orange; n*_Alnus_* = 67, n*_Vitis_* = 66, n_Control_ = 20) on two different test plants (*Alnus glutinosa* and *Vitis vinifera*) and nonplant control. Confidence intervals of 95% are depicted by transparency. Censored data are marked with a plus (+). *p*-values < 0.05 represent significant differences between insect species according to Log-rank test with Bonferroni correction. Dashed black line indicates median survival time (i.e., time when 50% of the insects are still alive).

Figure 1 illustrates the survival probability of both taxa, *Allygus* spp. and *O. ishidae*, on the two test plants *A. glutinosa* and *V. vinifera*, compared to a negative control without food source. Regarding the test plants no relevant difference occurred between the survival of *Allygus* spp. on alder compared to grapevine (*p* < 0.05), while *O. ishidae* performed statistically significantly better on alder than on grapevine (*p* < 0.001). When comparing the insect species, *O. ishidae* showed a significantly longer survival probability on alder compared to *Allygus* spp. (Figure 1, left). Conversely, the survival of *Allygus* spp. was significantly better on grapevine than that of *O. ishidae* (Figure 1, middle). None of the specimens could survive without any food source for more than 3 days (Figure 1, right). 

The influence of the sex of the individuals on the survival probability was also examined (Figure S3, Supplementary Materials). With the exception of the species *O. ishidae* on alder, females exhibited a higher survival probability than males.

**Figure 2:**
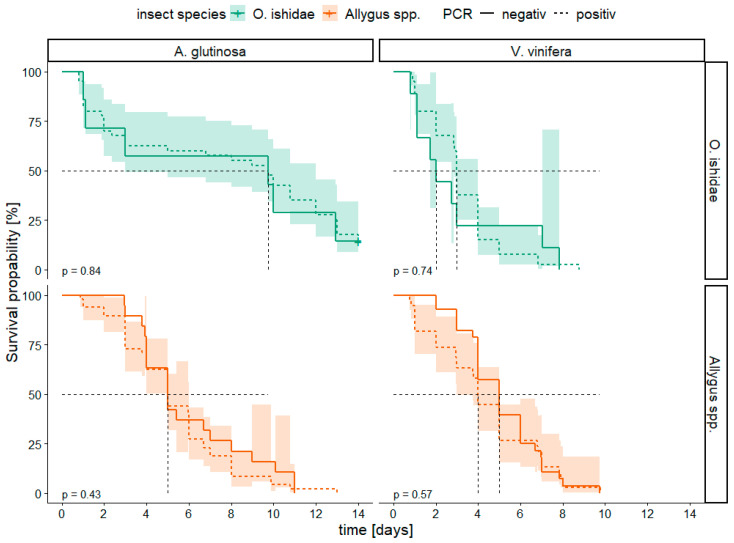
Kaplan–Meier survival curves for phytoplasma positive (+) and negative (−) tested *Orientus ishidae* (green; n*_Alnus+_* = 40, n*_Vitis+_* = 40, n*_Alnus−_* = 7, n*_Vitis−_* = 9) and *Allygus* spp. (orange; n*_Alnus+_* = 48, n*_Vitis+_* = 38, n*_Alnus−_*= 19, n*_Vitis−_* = 28) on two different test plants (*Alnus glutinosa* and *Vitis vinifera*). Infestation status was determined by PCR with 16SrV group specific primers. Confidence intervals of 95% are reported by transparency. Censored data are marked with a plus (+). *p*-values < 0.05 represent significant differences between PCR positive and negative insects according to Log-rank test with Bonferroni correction. Dashed black line indicates median survival time (i.e., time when 50% of the insects are still alive).

The effect of the phytoplasma infection status of the insects (infected compared to non-infected) on their survival probability was analyzed for both species. Single insect-single plant trials conducted in 2019 and 2020 revealed that the infection status did not significantly impair the survival probability of any species tested in our study (*p* > 0.05, Figure 2). 

**Figure 3:**
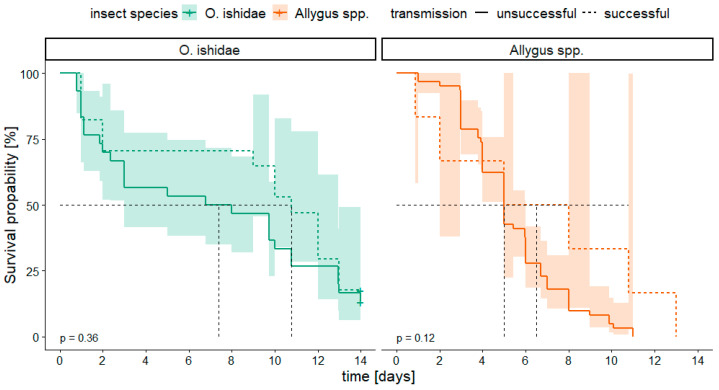
Kaplan–Meier survival curves of *Orientus ishidae* (green; n*_succ_* = 17, n*_unsucc_* = 30) and *Allygus* spp. (orange; n*_succ_* = 6, n*_unsucc+_* = 61) related to the transmission success of infected individuals to the test plant *Alnus glutinosa*. Confidence intervals of 95% are reported by transparency. Censored data are marked with a plus (+). *p*-values < 0.05 represent significant differences between PCR positive and negative insects according to Log-rank test with Bonferroni correction. Dashed black line indicates median survival time (i.e., time when 50% of the insects are still alive).

It was also investigated whether the inoculation success was influenced by the longevity of the insects on the test plants. Therefore, the survival of infected individuals was analyzed in relation to their ability to inoculate alder seedlings. While the dataset for transmission to *V. vinifera* was too limited for statistical analysis (five transmissions only), in the case of alder, we observed no effect of the longevity on the inoculation success, since there was no significant difference in the survival probability between insects that transmitted the phytoplasma successfully and those that did not (Figure 3). 

### 3.5. Phytoplasma Quantification

**Figure 4:**
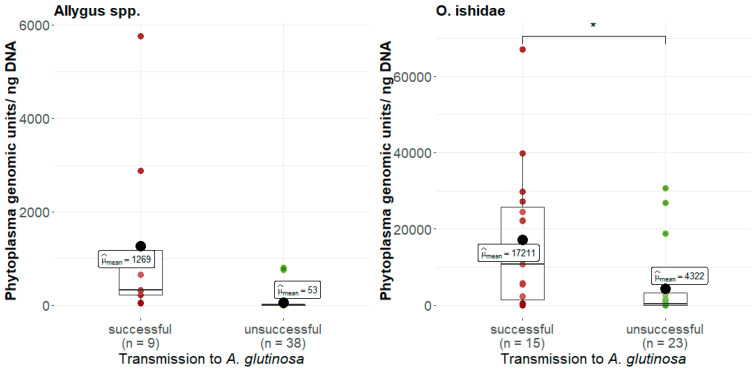
Comparison of the mean phytoplasma load (μ_mean_) of *Allygus* spp. and *Orientus ishidae* which resulted in a successful (red) or unsuccessful transmission (green) to *Alnus glutinosa*. Statistically significant differences between successful and unsuccessful transmission to the test plant was estimated by Welch’s *t*-Test (* *p* < 0.05).

The phytoplasma load was quantified in all infected specimens, and it was also differentiated among individuals, which transmitted successfully and those that did not. In both species, insects that successfully transmitted had a significantly higher titer compared to infected specimens without transmission success (Figure 4). Single *O. ishidae* generally had a titer approximately 10 times higher than *Allygus* spp. (see scaling in Figure 4). Based on the quantitative data, it was possible to define a minimum inoculation titer (MIT) for transmission to alder for both species. The MIT was determined from the lowest titer of *Allygus* spp. or *O. ishidae* individuals that still successfully transmitted. The titer of successfully transmitting *Allygus* spp. was at least 62 phytoplasma genomic units/ng DNA. Single *O. ishidae* transmitted at a minimum titer of 218 phytoplasma genomic units/ng DNA. Individuals with a phytoplasma load above the species-specific MIT were therefore classified as potentially infective. All of the four *O. ishidae* specimens that successfully inoculated *V. vinifera* in the single insect-single plant trials had a high phytoplasma load.

Based on the MIT, the transmission success was examined in relation to the inoculation access period (IAP) of potentially infective insects. The results, summarized in Table 4, illustrate that *O. ishidae* is able to successfully transmit to alder within only one day after inoculation. In contrast, for *Allygus* spp., no individual reached the MIT after one day, but they exceeded the MIT after two days, followed by successful transmission to alder. A majority of the insects (*Allygus* spp. 78% and *O. ishidae* 75%) classified as infective transmitted the phytoplasma after an extended IAP of up to 10 days.

**Figure 5:**
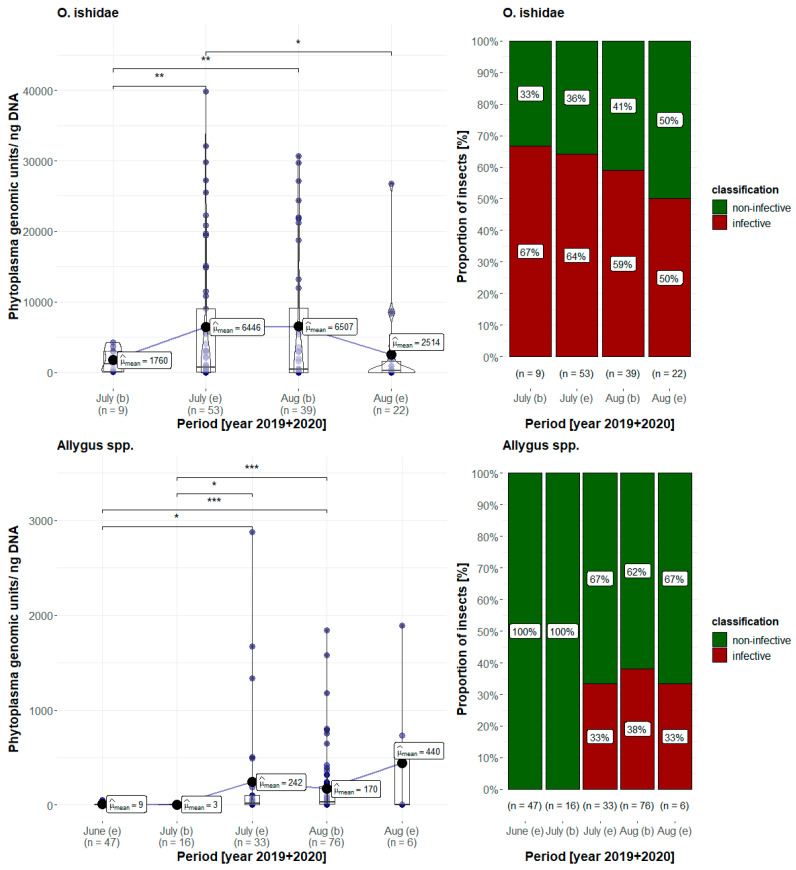
(Left) Comparison of the estimated mean phytoplasma load (μ_mean_) of (top) *Orientus ishidae* and (bottom) *Allygus* spp. between the beginning (=b) of June and the end (=e) of August (data pooled from 2019 and 2020). Statistically significant differences between periods were estimated by Welch’s ANOVA (* *p* < 0.05, ** *p* < 0.01 and *** *p* < 0.001). (Right) Proportion of *Orientus ishidae* (top) and *Allygus* spp. (bottom) classified as infective (red) and noninfective (green) during the season from July to August (data pooled from 2019 and 2020).

Furthermore, the variation of the phytoplasma load of field-collected leafhoppers of both taxa during the season and the proportion of specimens classified as infective (having a phytoplasma load exceeding MIT) was calculated and related to the collection time (Figure 5, note the different scaling between species). Interestingly, the trend for both species is different. In *Allygus* spp., the phytoplasma load steadily increased from June to July, with individuals reaching the MIT not before the end of July. In contrast, *O. ishidae* specimens were already classified as infective from the first sampling date at the end of June. However, it is worth noting that even though *O. ishidae* were already infective at the beginning of the season, their phytoplasma load showed high variability. 

### 3.6. Genotyping of Phytoplasmas in Insects and Plants

The phytoplasma genotypes in infected leafhoppers and test plants were determined based on the gene markers *map* and *vmp*. As shown in Table 5, the *map* type M38, which belongs to the cluster FD2, is predominant in both insect species and in both test plant species. One alder seedling became infected by the PGY genotype M48 after inoculation with *Allygus* spp. While *Allygus* spp. carried different *map* PGY genotypes, *O. ishidae* was almost exclusively loaded with the M38 genotype, the same genotype that was also transmitted to *V. vinifera* by this species in the transmission trials. The differentiation on the *vmp*A gene confirmed the expected allocation of all PGY-associated *map* genotypes to the VmpA-I cluster and the *map* genotype M38 to the VmpA-II cluster.

## 4. Discussion

The European origin of FD phytoplasmas is well documented [11]. In particular, alder trees are considered a main source of FD phytoplasmas. Rigamonti et al. [12] reported infection rates of black alder in northwestern Italy of about 91%, which fits well with infection rates in Germany or France [11,18]. While the presence of *S. titanus,* the main vector of “flavescence dorée” phytoplasmas, is the prerequisite for FD outbreaks in vineyards, autochthonous Deltocephalinae leafhoppers may occasionally transmit “flavescence dorée” phytoplasmas from alder to grapevine, thereby establishing sources of inoculum in the vineyards that could increase the risk of outbreaks once *S. titanus* emerges. However, the probability and the parameters of “flavescence dorée” phytoplasma transmission from alder to grapevine by Deltocephalinae species remain poorly understood, although this information is required for risk analysis, particularly for regions where *S. titanus* is approaching. Since *Allygus* spp. and *O. ishidae* are confirmed vectors of “flavescence dorée” phytoplasmas to alder under experimental conditions [11,20], this study focused on the transmission parameters of field-captured adults of these two taxa in transmission trials to *V. vinifera*. Malembic-Maher et al. [11] reported high infection rates by 16SrV phytoplasmas in the Deltocephalinae *Allygus* spp., *O. ishidae* and the Macropsinae *Oncopsis alni.* Only the Deltocephalinae transmitted “flavescence dorée” phytoplasmas, while *O. alni* transmitted PGY strains that are not transmitted by *S. titanus*.

The objective of this study was to elaborate if *Allygus* spp. and *O. ishidae,* collected from infected alder in the field, were able to infect grapevine and to elucidate the transmission parameters to assess the risk for FD outbreaks. As both species are not closely associated with grapevine and were only occasionally captured in vineyards, we tested three different experimental designs of transmission assays. First, dual choice experiments with both, the preferred host plant alder and the occasional host grapevine in the same cage, allowed the leafhoppers to move freely between the natural host and grapevine. Second, transfer trials where field-captured insects are adapted on alder before being forced to feed on grapevine. Third, a one-by-one assignment between single leafhoppers on their host alder and the occasional grapevine with three objectives: (i) to ensure feeding of the test insects on the plant, (ii) to record the individual survival time and (iii) to correlate transmission success with the individual insect’s phytoplasma titers. 

The experimental set-ups of the transmission trials were adapted from our previous studies with *O. alni*, *Allygus* spp. and *O. ishidae* using groups of insects [11,13,19]. The data from all three experimental set-ups confirmed previous results, demonstrating that both *Allygus* spp. and *O. ishidae* efficiently inoculated alder seedlings, although transmission rates were lower in the third experiment where test plants were inoculated by only one insect vector. The transmission rates of *Allygus* spp. to alder (29–80%) were much higher than the 20% previously reported by Malembic-Maher et al. [11], while *O. ishidae* was confirmed as a very efficient vector of 16SrV phytoplasmas with transmission rates ranging from 67 to 100% in all studies. Thus, both taxa play a role in maintaining 16SrV phytoplasmas in European alder populations. 

While larger hot-water treated grapevine cuttings cv. Chardonnay were used for the dual choice and transfer approaches with groups of insects, the single insect-single plan3 experiments were carried out with smaller ex vitro plants cv. Scheurebe. It is conceivable that the smaller plantlets with more delicate tissue react more sensitively to inoculation and, thus, the transmission success was higher. 

Transmission to grapevine was only achieved by *O. ishidae* in the first (one successful transmission) and third (four successful transmissions) experimental set-ups, with comparable transmission rates of 17% and 13%, respectively. The general capability of *O. ishidae* to inoculate grapevine plantlets under forced experimental conditions had been previously demonstrated by Lessio et al. [9]. However, they were only successful in two trials with specimens that performed an acquisition access period (AAP) on infected plants under experimental conditions. We obtained successful inoculation of grapevine with naturally infected *O. ishidae* and proved that *O. ishidae* is able to transmit “flavescence dorée” phytoplasma from alder to grapevine, since the FD *map* genotype M38 was detected in both the successful vectors and the recipient grapevine plants. According to Malembic-Maher et al. [11] *map* genotype M38 is identical to grapevine map-FD2 genotypes detected in FD outbreaks and can be transmitted by *S. titanus.*

As it was worked with field-captured adults at different capture periods, infection rates of insects and transmission rates varied among the years of experimentation. In the transmission trails with dual choice and by transfer, infection rates of *Allygus* spp. and *O. ishidae* ranged from 14 to 72% and 32 to 80%, respectively, confirming the data presented by Malembic-Maher et al. [11], who found 60% infected *Allygus* spp. and 52% infected *O. ishidae*. Comparable infection rates for *O. ishidae* were reported from Slovenia [37] (25–50%), Switzerland [38] (85%) and the Alsace region in France [39] (56%). 

The single insect-single plant approach allowed us to analyze the correlation between survival probability, infection status of the individuals and transmission success. The statistical analysis revealed a significantly higher survival probability of *O. ishidae* on alder compared to grapevine, indicating that alder is a true host plant for *O. ishidae*. Both species are more or less polyphagous with adults preferring some trees and shrubs [9,40,41,42,43]. An important difference between both taxa is the ability of *O. ishidae* to complete its whole life cycle on alder, whereas adult *Allygus* spp. move to alder after their larval instars have fed on herbaceous vegetation [44]. This observation was supported by recent data from Rizzoli et al. [38], who demonstrated that *O. ishidae* is able to complete its biological cycle on *Alnus glutinosa* and could acquire 16SrV phytoplasmas efficiently from alder. Accordingly, the low survival probability of both taxa on *Vitis vinifera* confirms the hypothesis that grapevine is not very attractive as food source for both species. The generally high transmission rates to alder indicate that both vector species can feed on alder in the adult stage. The survival times of *Allygus* spp. and *O. ishidae* on *V. vinifera* exceeded that of nonplant controls, which indicates that both species fed on grapevine recipient plants but were restricted in their ability to transmit the pathogen to these test plants. Additional electrical penetration graph (EPG) studies would be useful to understand and characterize the feeding mechanism underlying the varying transmission success between these species. Regarding FD, the feeding characteristics were only described for the vector *S. titanus* [45].

The survival probability was similar for transmitters and nontransmitters of both species and the infection status did not influence their survival, as it has been also demonstrated for infected *S. titanus* [46]. Reports on a reduced fitness of infected phytoplasma vectors, e.g., *Macrosteles quadripunctulatus* in the Chrysanthemun yellows model [47], showed that this is not always the case. 

If the infection status does not affect the survival or fitness of the test insects, other parameters must be considered to explain the transmission success. The quantification of the phytoplasma load in all infected individuals revealed a significant difference in the phytoplasma titers of successfully transmitting specimens of both taxa, which allowed us to define a minimum inoculation titer (MIT) for transmission. As phytoplasmas have to multiply in the salivary glands of the insect during the latency period (LP) to render an individual infective, the phytoplasma load of an insect distinguishes between noninfective individuals that just acquired the phytoplasmas and infective individuals that can successfully transmit the phytoplasma with the saliva to the plant [48,49].

Interestingly, not only did *O. ishidae* have a 3-fold higher MIT than *Allygus* spp. but infective *O. ishidae* also generally had much higher phytoplasma loads, ranging predominantly from 10^3^ to 10^4^ phytoplasma genomic units (GU) per ng DNA, compared to infective *Allygus* spp. individuals with titers of 10^2^ to 10^3^ phytoplasma GU per ng DNA. However, the data are in the range of phytoplasma titers reported for *Macrosteles quadripunctulatus* (10^4^ phytoplasma GU per ng total DNA) and *Euscelidius variegatus* (10^3^ phytoplasma GU per ng total DNA) in the Chrysanthemun yellows model [47,50]. 

Only *O. ishidae* was able to transmit successfully to grapevine, and all these individuals had a high phytoplasma titer (10^4^ phytoplasmas GU per ng DNA), a level that was never achieved in individuals of *Allygus* spp. From this observation, it can be assumed that the threshold titer for successful inoculation of grapevine might be higher than the MIT for alder. 

The majority of individuals that reached the MIT successfully transmitted the pathogen to alder seedlings. The transmission probability generally increased with longer IAP, but *O. ishidae* succeeded in inoculating alder seedlings with an IAP of at least one day. In the case of *Allygus* spp., an IAP of at least two days resulted in transmission to alder, while a minimum of one day could not be tested in our studies. *O. ishidae* did not survive longer than 4 days on grapevine, but this was sufficient to transfer the agent to the plantlets. It can be assumed that highly infective individuals do not require a long incubation period on small plantlets as used for the transmission trials. 

The differences between both taxa in terms of infection rate, incubation time and transmission success may be related to their different biological cycle [9,40,41,42,43]. Tracking the evolution of the mean phytoplasma titer and the percentage of infective individuals during the season, it was observed that already the first captured adults of *O. ishidae* showed a high phytoplasma load and the percentage of infective individuals remained at a high level throughout the entire season. The first instars of *O. ishidae* have the opportunity to acquire the pathogen from infected alder and the latency period can be completed by the end of nymphal development. In contrast, *Allygus* spp. only reached a substantial phytoplasma titer in the middle of the season, with the percentage decreasing toward the end. This corresponds with the life cycle of *Allygus* spp., which has the ability to acquire 16SrV phytoplasmas only when the adults move from the soil vegetation to the infected alder trees. A suspected latency period of four weeks thereafter could explain the observed low phytoplasma titers in *Allygus* spp. until mid-July. Therefore, it can be concluded that *O. ishidae* has a higher potential to acquire “flavescence dorée” phytoplasmas early from alder and to reach phytoplasma titers more relevant for transmission to other plants. In consequence, infective individuals of *O. ishidae* are present in the vicinity of vineyards for a much longer period than infective individuals of *Allygus* spp. increasing the probability of accidental “flavescence dorée” phytoplasma transmission to grapevine. 

In 2017, in Alsace, a single grapevine infected with *map* genotype M38 was detected at a site where *S. titanus* was still absent [39]. The presence of *Allygus* spp. and *O. ishidae* infected by the M38 strain at the site led to the conclusion that these alternative vectors might be responsible for the transfer of M38 from reservoir alders to grapevine. A similar scenario occurred in Germany in 2020, with the detection of a M38 infected grapevine in a commercial vineyard in Rhineland-Palatinate, adjacent to an alder stand where specimens of *Allygus* and *O. ishidae* infected by M38 were sampled [19]. This study supports the conclusions by Malembic-Maher et al. [11] and Rizzoli et al. [38] that the coincidence of infected alder with *Allygus spp*. or *O. ishidae* and nearby vineyards may provoke the emergence of isolated FD cases that could trigger FD spread once *S. titanus* becomes established. 

Especially in Germany, which is still assessed as FD-free but where the nonepidemic PGY variants exhibit the same symptoms as “flavescence dorée” phytoplasmas, the precise identification of the specific phytoplasma strain is crucial. Analyses of the phytoplasma genotypes in insects and test plants based on the housekeeping genes *map* and *vmp* showed that in all test plants as well as in almost all *O. ishidae* only *map* genotype M38 belonging to the genetic VmpA-II cluster was found. M38 was also the dominant genotype in infected *Allygus* spp. However, in Switzerland, Rizzoli et al. [38], and in northwestern Italy, Rigamonti et al. [12], evidenced a high prevalence of the *map* genotype M50 in *O. ishidae* as well as in alder trees. Similarly, Malembic-Maher et al. [11] obtained a high percentage of transmissions by *O. ishidae* with *map* genotype M50 originating from southwestern France. As M38 and M50 belong to different FD clusters, it might be concluded that the map-FD1 type M50 is more prevalent in regions south and west of the Alps whereas map-FD2 type M38 is dominant in regions north of the Alps. It remains to be elucidated what this means for the epidemiology of FD.

These data prove the possibility of “flavescence dorée” phytoplasma transmission from alder to grapevine. It may be considered a rare event, as transmissions from alder to grapevine under field conditions are likely less efficient than in our laboratory studies. However, the further spread of the invasive species *O. ishidae* could lead to a higher prevalence of M38 in alder, since it is shown in this study that *O. ishidae* is an efficient vector of the M38 strain from alder to alder. Preliminary data from Germany support this hypothesis [18] and demonstrate that alder trees are widespread in the vicinity of vineyards. This aspect might be similar in other viticultural regions with comparable natural habitats, e.g., Switzerland [38] and France [39]. Since it is of great importance for FD-free regions like Germany to identify and eradicate single FD-infected grapevines before the arrival of the vector *S. titanus*, monitoring should be focused on vineyards in the vicinity of alder stands. For this reason, currently risk maps of vineyards in the vicinity of alder stands are under preparation [18].

## 5. Conclusions 

This study aimed to evaluate the risk posed by two Deltocephalinae leafhopper taxa for the spread of “flavescence dorée” from naturally infected alder into vineyards. Populations of *Allygus* spp. and *Orientus ishidae* collected from infected alder trees in southwestern Germany were infected by “flavescence dorée” phytoplasmas and readily transmitted the phytoplasma to alder seedlings, which confirms the results of previous studies. One to two days of inoculation access were sufficient for successful transmission to alder. Although both species survived and presumably fed on grapevine for several days, only *O. ishidae* transmitted “flavescence dorée” phytoplasmas successfully to grapevine in two of three modes of transmission trials, although with less efficiency compared to alder. In addition, *O. ishidae* carries higher titers of “flavescence dorée” phytoplasmas compared to *Allygus* spp. during a longer period within the season. This leads to the conclusion that the invasive leafhopper *O. ishidae* poses a higher risk for disseminating “flavescence dorée” phytoplasmas to vineyards than the two autochthonous *Allygus* species present on alder. However, the probability of *O. ishidae* spreading “flavescence dorée” phytoplasmas from alder to vineyards appears to be low, due to the feeding biology of this species and rather low transmission rates to grapevine even in controlled transmission trials. Nevertheless, every single grapevine infected by “flavescence dorée” phytoplasmas poses a risk of FD outbreaks in the presence of the vector *S. titanus*. Therefore, specific monitoring strategies of vineyards adjacent to alder stands need to be established in FD-free regions to prevent FD outbreaks.

## Figures and Tables

**Table 1:** Phytoplasma transmission rate and infectivity of *Allygus* spp. and *Orientus ishidae* on test plants *Alnus glutinosa* and *Vitis vinifera* in dual choice experiments in years 2017 and 2018.

**2017**	**number of plants PCR+/** **number of plants tested**	**number of insects caged**	**number of insects PCR+/** **number of insects tested**
*Allygus* spp.	-	36	14/28 (50%)
*A. glutinosa*	2/4 (50%)	**-**	**-**
*V. vinifera*	0/4 (0%)	**-**	**-**
*O. ishidae*	-	41	13/26 (50%)
*A. glutinosa*	5/6 (83%)	-	-
*V. vinifera*	1/6 (17%)	-	-
**2018**	**number of plants PCR+/** **number of plants tested**	**number of insects caged**	**number of insects PCR+/number of insects tested**
*Allygus* spp.	**-**	23	6/19 (32%)
*A. glutinosa*	1/2 (50%)	-	-
*V. vinifera*	0/2 (0%)	-	-
*O. ishidae*	-	52	14/44 (32%)
*A. glutinosa*	1/4 (25%)	-	-
*V. vinifera*	0/3 (0%)	-	-

**Table 2:** Phytoplasma transmission rates and infectivity of *Allygus* spp. and *Orientus ishidae* in transfer trials from *Alnus glutinosa* to *Vitis vinifera* in the years 2017 to 2019.

**2017**	**number of plants PCR+/** **number of plants tested**	**number of insects caged**	**number of insects PCR+/** **number of insects tested**
1st test plant *A. glutinosa*			
*Allygus* spp.	4/5 (80%)	42	7/15 (47%)
*O. ishidae*	5/5 (100%)	85	15/18 (83%)
Transfer to 2nd test plant *V. vinifera*			
*Allygus* spp.	0/5 (0%)	27	14/27 (52%)
*O. ishidae*	0/5 (0%)	67	48/60 (80%)
**2018**	**number of plants PCR+/** **number of plants tested**	**number of insects caged**	**number of insects PCR+/** **number of insects tested**
1st test plant *A. glutinosa*			
*Allygus* spp.	3/7 (43%)	30	3/8 (38%)
*O. ishidae*	8/12 (67%)	94	14/25 (56%)
Transfer to 2nd test plant *V. vinifera*			
*Allygus* spp.	0/7 (0%)	19	8/15 (53%)
*O. ishidae*	0/12 (0%)	64	27/55 (49%)
**2019**	**number of plants PCR+/** **number of plants tested**	**number of insects caged**	**number of insects PCR+/** **number of insects tested**
1st test plant *A. glutinosa*			
*Allygus* spp.	2/7 (29%)	55	13/18 (72%)
*O. ishidae*	11/14 (79%)	152	15/38 (39%)
Transfer to 2nd test plant *V. vinifera*			
*Allygus* spp.	0/7 (0%)	36	4/29 (14%)
*O. ishidae*	0/14 (0%)	114	51/113 (45%)

**Table 3:** Phytoplasma transmission rates and infectivity of *Allygus* spp. and *Orientus ishidae* in single insect-single plant trials with *Alnus glutinosa* or *Vitis vinifera* in the years 2019 and 2020.

**2019**	**number of plants PCR+/** **number of plants tested**	**number of insects PCR+/** **number of insects tested**
*A. glutinosa*		
*Allygus* spp.	2/17 (12%)	11/17 (65%)
*O. ishidae*	1/18 (16%)	14/18 (78%)
*V. vinifera*		
*Allygus* spp.	0/17 (0%)	12/17 (71%)
*O. ishidae*	0/19 (0%)	12/19 (63%)
**2020**	**number of plants PCR+/** **number of plants tested**	**number of insects PCR+/** **number of insects tested**
*A. glutinosa*		
*Allygus* spp.	7/50 (14%)	36/50 (72%)
*O.ishidae*	13/30 (43%)	28/30 (93%)
*V. vinifera*		
*Allygus* spp.	0/49 (0%)	24/49 (49%)
*O. ishidae*	4/30 (13%)	26/30 (87%)

**Table 4:** Phytoplasma transmission rates to *Alnus glutinosa* seedlings by (a) *Allygus* spp and (b) *Orientus ishidae* with varying inoculation access periods (IAP).

IAP in Days	Number of Trials with Infected Insects	Number of Infective Insects	Number of Transmissions	Transmission Rate of Infective Insects
(a) *Allygus* spp.				
1	2	0	0	-
2	2	1	1	100%
3	6	0	0	-
4	4	1	0	0%
5	9	2	2	100%
6–8	13	4	3	75%
8–10	0	0	0	-
>10	1	1	1	100%
(b) *O. ishidae*				
1	5	4	2	50%
2	3	2	2	100%
3	0	0	0	-
4	0	0	0	-
5	1	1	0	0%
6–8	1	1	0	0%
8–10	3	3	3	100%
>10	13	9	8	89%

**Table 5:** Genotyping of phytoplasma infected insects *Allygus* spp. and *Orientus ishidae* and the respective test plants *Alnus glutinosa* and *Vitis vinifera* based on the *map* gene.

		Map Genotypes						
		FD2	PGY					
Species	Number Analyzed	M38	M14	M39	M47	M48	M53	M110
*Allygus* spp.	65	56	2	1	2	1	2	1
*O. ishidae*	189	188				1		
*A. glutinosa*	40	39				1		
*V. vinifera*	5	5						

## Data Availability

Data are included in the article or Supplementary Materials.

## Supplementary Materials

The following supporting information can be downloaded at: https://www.mdpi.com/article/10.3390/microorganisms11112766/s1, Figure S1: (a) Schematic diagram for a dual choice approach with several test plants in one cage. (b) Schematic diagram for a transmission trial assay with transfer among test plants. Figure S2: Symptom expression of an infected grapevine plantlet. Figure S3: Kaplan–Meier survival curves for females and males of *Orientus ishidae* and *Allygus* spp.
