# Supplementary material for: Risk Assessment for the Spread of Flavescence Dorée-Related Phytoplasmas from Alder to Grapevine by Alternative Insect Vectors in Germany
Source: Microorganisms. 2023 Nov 14;11(11):2766. doi: 10.3390/microorganisms11112766 (PMC10673178; doi:10.3390/microorganisms11112766)
Supplement: Supplementary file 1 [file microorganisms-11-02766-s001.zip › microorganisms-2667333-supplementary.pdf]

Supplementary Materials

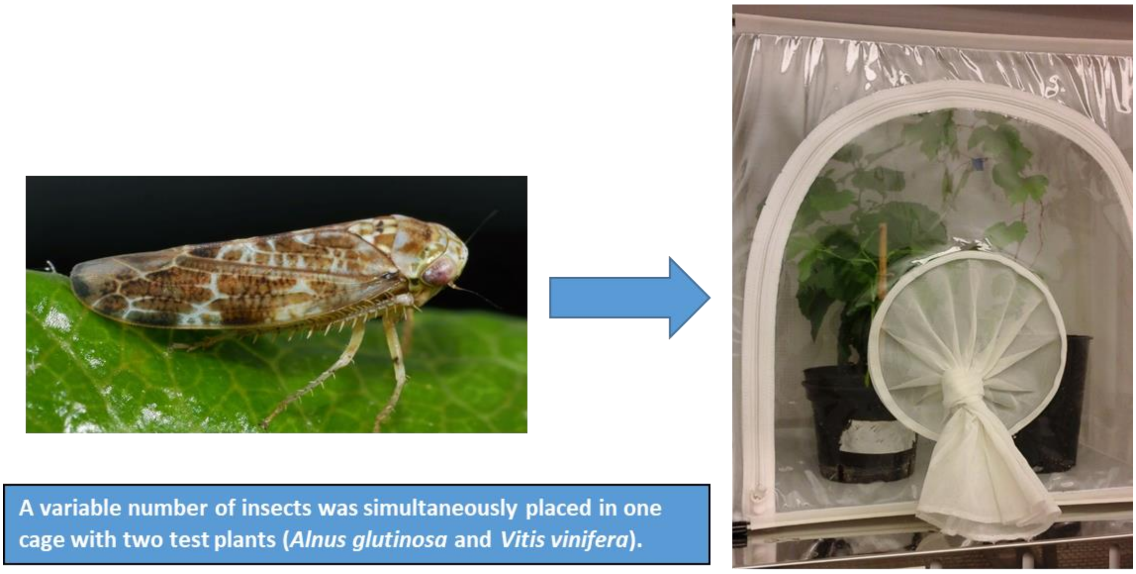

Figure S1a. Schematic diagram for a dual choice approach with stwo test plants in the same cage with specimens of *Allygus spp*. or *O. ishidae*, respectively. Dead specimens were sporadically removed from the cage and frozen at -20°C.

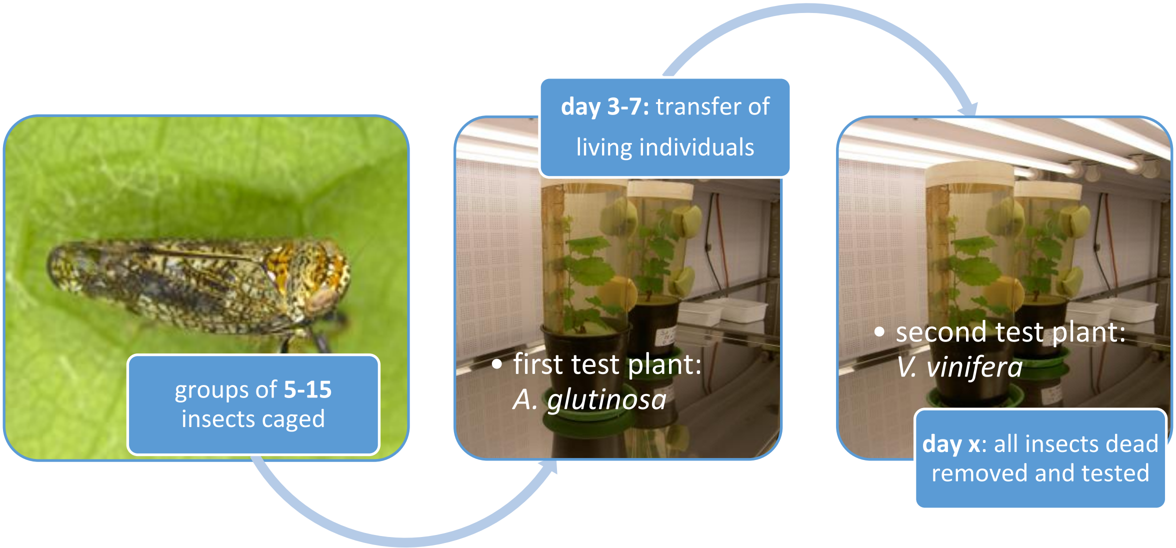

Figure S1b. Schematic diagram for a transmission trial assay with transfer of *Allygus spp*. or *O. ishidae* specimens, respectively, among test plants *A. glutinosa* and *V. vinifera*. Dead specimens were sporadically removed from the cage and frozen at -20°C.

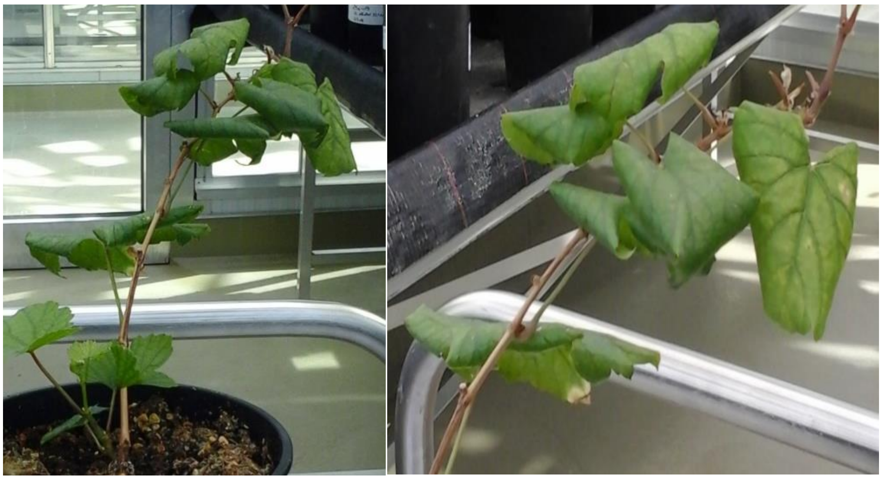

Figure S2. Symptom expression of a grapevine plantlet (cultivar 'Scheurebe') three months after inoculation with naturally infected *Orientus ishidae* under controlled conditions.

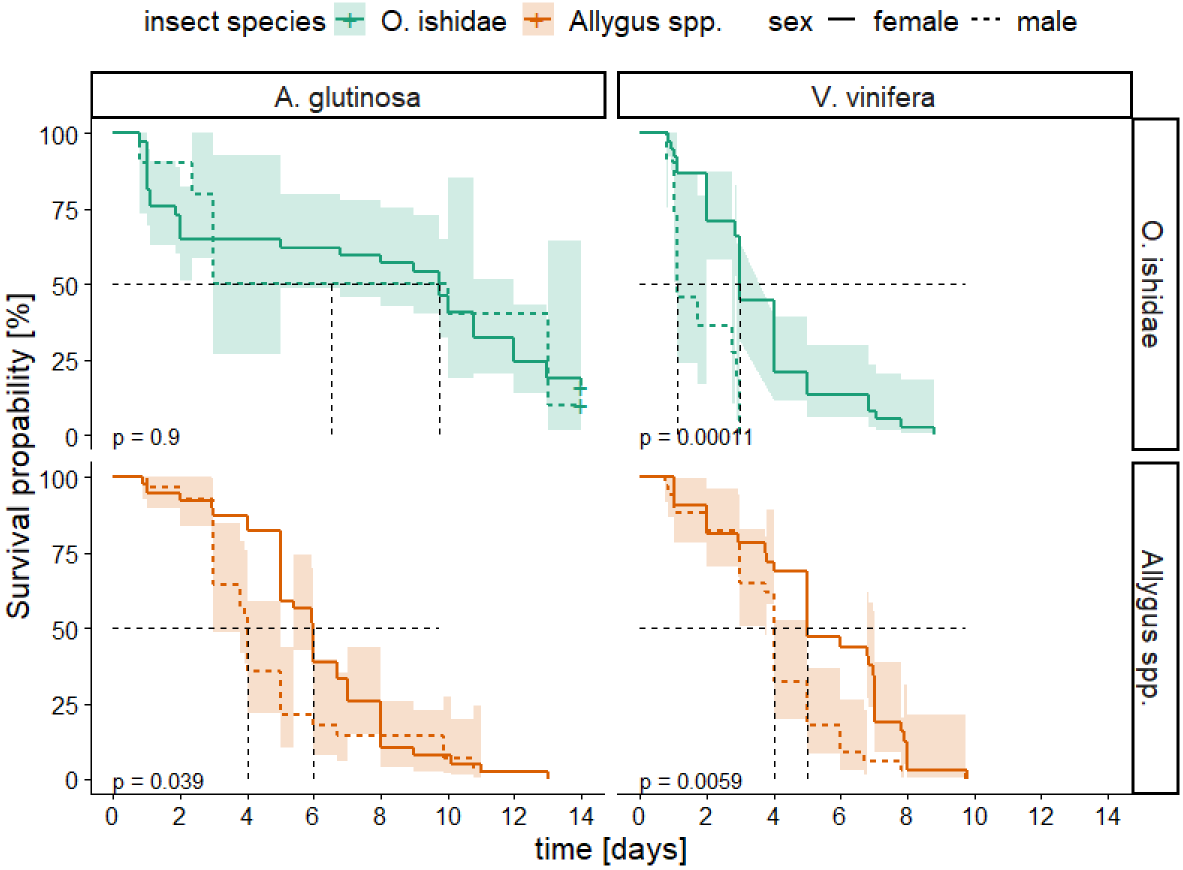

Figure S3. Kaplan-Meier survival curves for *Orientus ishidae* (top, green) and *Allygus* spp. (orange; buttom) females (continuous line) and males (dashed line) on two different test plants: (left) *Alnus glutinosa* ($n_{orientus\_females}$= 37, $n_{orientus\_males}$= 10, $n_{allyguss\_females}$= 39, $n_{allygus\_males}$= 28) and (right) *Vitis vinifera* ($n_{orientus\_females}$= 38, $n_{orientus\_males}$= 11, $n_{allyguss\_females}$= 32, $n_{allygus\_males}$= 34). 95% confidence intervals are reported by transparency. Censored data are marked with a plus (+). P-values<0.05 represent significant differences between insect sexes according to Log-rank test with Bonferroni correction. Dashed black line indicate median survival time (i.e., time when 50% of the insects are still alive).
